# Engineered nanovesicles from stromal vascular fraction promote angiogenesis and adipogenesis inside decellularized adipose tissue through encapsulating growth factors

**DOI:** 10.1038/s41598-022-27176-w

**Published:** 2023-01-13

**Authors:** Jun Tu, Yuyang Zeng, Ran An, Jiaming Sun, Huicai Wen

**Affiliations:** 1 Department of Plastic, Medical Center of Burn Plastic and Wound Repair, The First Affiliated Hospital of Nanchang University, Nanchang University, Nanchang, China; 2 Department of Dermatology, Tongji Hospital, Tongji Medical College, Huazhong University of Science and Technology, Wuhan, China; 3 Department of Plastic Surgery, Union Hospital, Tongji Medical College, Huazhong University of Science and Technology, Wuhan, China

**Keywords:** Nanoparticles, Regenerative medicine

## Abstract

Acellular matrix is a commonly used biomaterial in the field of biomedical engineering and revascularization is the key process to affect the effect of acellular matrix on tissue regeneration. The application of bioactive factors related to angiogenesis has been popular in the regulation of revascularization, but the immune system clearance, uncontrollable systemic reactions, and other factors make this method face challenges. Recent reports showed that engineered cells into nanovesicles can reorganize cell membranes and encapsulate cellular active factors, extending the in vitro preservation of cytokines. However, the problems of exogenous biological contamination and tumorigenicity restricted the clinical transformation and wide application of this method. Here, we for the first time engineer stromal vascular fraction (SVF) which is extracted from fat into nanovesicles (SVF-EVs) for angiogenesis in the acellular matrix. SVF-EVs not only promote the migration of vascular endothelial cells in vitro, but also facilitate the lipogenic differentiation of mesenchymal stem cells. In vivo, SVF-EVs enhanced the retention of decellularized adipose tissue after transplanting to the subcutaneous area of nude mice. Immunofluorescence staining further showed that SVF-EVs promoted the formation of vascular networks with large lumen diameter in the grafted acellular matrix, accompanied by adipocyte regeneration peripherally. These findings reveal that SVF-EVs can be a viable method for accelerating revascularization in acellular matrix, and this process of squeezing tissue into nanovesicles shows the potential for rapid clinical transformation.

## Introduction

Acellular matrix has been used since the beginning of the 1990s to resolve some of the issues associated with insufficient sources of autologous materials and suboptimal bioactivity of synthetic materials, and it is widely used in clinical practice for filling tissue defects, trauma repair, and facial contour remodeling^1–4^. Revascularization of the acellular matrix is a key issue that affects the outcome of therapeutic procedures^5,6^. During preparation, the acellular matrix is completely stripped off the vascular endothelial cells, and even the walls of some fine vessels are damaged to varying degrees^7^. The acellular matrix loses its original vascular network and requires revascularization to achieve blood perfusion after implantation in the body. However, the inflammation, hypoxic state, and lack of growth factors at the implantation site are often not conducive to revascularization, which in turn affects the effectiveness of acellular matrix repair of the defect^8,9^. How to promote the angiogenic process of acellular matrix has become one of the research hotspots in regenerative medicine^10^.

Many studies on vascularization have identified a set of growth factors and cytokines that act sequentially to cause vascular endothelial cell migration and morphological changes, culminating in forming a connected vascular network within the tissue^11,12^. The identified growth factors include Angiopoietin (Ang), hypoxia inducible factor (HIF), vascular endothelial growth factor (VEGF), fibroblast growth factor (FGF), and so on, which have been used to vascularize grafts or biomaterials^13–17^. Nevertheless, owing to the short half-life caused by direct host-facing immune system clearance, bioactive factors are depleted before forming an effective vascular network^18^. These factors may be involved in other regulating pathways that can cause metabolic disturbances, structural abnormalities, and even tumorigenic effects, limiting the use of growth factors to promote vascularization in the acellular matrix^19,20^. In recent years, cell product like nanovesicles, containing growth factors shows lower healthcare risk and systemic reaction, leading to good application potential and research value^21–23^.

Extracellular vesicles are nanoscale biological products secreted by cells that transport biological information between cells and the extracellular matrix^24^. They can contain various bioactive substances, including proteins, mRNAs, miRNAs, and other components^25^. These components can be preserved for a longer time and exert biological activity owing to the protective effect of the phospholipid envelope of extracellular vesicles^26^. Studies have revealed that extracellular vesicles derived from vascular endothelial cells, pericytes, and mesenchymal stem cells exist promising pro-angiogenic effects^27,28^. However, natural extracellular vesicles are exclusively secreted by cells, and related study has demonstrated that each cell secretes very little vesicles per day^29^. Collecting these specific cell-generated extracellular vesicles from body fluids is time-consuming and resource-intensive. The artificial synthesis of nanovesicles, such as culturing special types of cells to obtain vesicles, and the formation of liposomes by water–oil interface interaction, has an effective formation rate and product consistency^30–33^. But the inevitable addition of drugs, reagents, and other substances kept it far from clinical application^34^.

Studies have depicted that mechanical shear can rupture cell membranes and that, in a suitable environment, fragmented segments of cell membranes exhibit mobility and reassemble into intact spherical envelopes using energy gained from ATP hydrolysis^35–37^. In 2017, Kim et al.^35^ obtained vesicles with 100–200 nm diameters after passing cells through filter membranes with different pore sizes. These vesicles, also known as “engineered vesicles”, can be obtained in significant numbers by simple physical pushing and squeezing and have been successfully used to transform erythrocytes and mesenchymal stem cells into active protein-encapsulated vesicles. The amount of encapsulated proteins and number of prepared vesicles also increased. The existing techniques for preparing engineered vesicles enable the transformation of cells into nanovesicles, but applying in vitro cultured cell in clinical practice still raises ethical concerns such as tumorigenicity and immunogenicity testing. Stromal vascular fractions (SVF) are bioactive components extracted from adipose tissue and contain endothelial cells, pericytes, mesenchymal stem cells, hematopoietic stem cells, and extracellular matrix, which are clinically useful tissue components^38^. Many studies have demonstrated that SVF promotes vascularization and has also been used in clinic to increase volume retention after autologous fat grafting^38,39^. We speculate that the engineered squeezing process has the potential to convert SVF into engineered nanovesicles and retain the bioactive effect of SVF. This promising product may enhance tissue regeneration and exhibit clinical availability similar to SVF, and the simple process makes it cost-effective for clinical use.

In this study, for the first time, we prepared biological tissue samples (not pure cells), SVF, into engineered nanovesicles (SVF-EVs) by the shear action of filter membranes with different pore sizes and co-transplanted them with readily available decellularized adipose tissue (DAT) to test their angiogenic effects. Transmission electron microscopy (TEM) and nanoparticle tracking analysis (NTA) were used to investigate the morphological characteristics of the engineered vesicles. Western blotting was subsequently used to study the content of pro-angiogenic proteins encapsulated within the vesicles. The effect of engineered vesicles on the cellular behavior of vascular endothelial cells and adipose mesenchymal stem cells was studied in vitro, and the effect of vesicles on vascularization of the acellular matrix was evaluated histologically.

## Methods

This study was approved by the Ethics Committee of the Wuhan Union Hospital, and all methods were carried out in accordance with relevant guidelines and regulations.

### Fabrication of SVF-EVs

Adipose tissue collected via liposuction from healthy female donors were obtained from the plastic surgery within Wuhan Union Hospital, and these donors signed informed consent forms for the study before surgery. Tissue samples were washed three times with sterile normal saline (Huaren Pharm., China) and centrifuged at 65×g for 5 min to remove solution. 10 mL adipose tissues were transferred into a 20-mL syringe which connected with another 20-mL syringe through a Luer-Lok taper, and then squeezed repeatedly within 1 min. The obtained suspension was centrifuged at 2000×g for 5 min. After centrifugation, the oil layer was discarded, the remaining portion was saved and the connective tissue remnants in it were picked out by fine needle. Subsequently, the obtained mixture was further ultrasonicated at 4 °C for 5 min and followed by centrifugation at 2000×g for 5 min. After processing, the floating cream layer was removed, the remaining fraction was subjected to sequentially extrusion five times through polycarbonate filter membranes with decreasing apertures (10 μm, 5 μm and 1 μm) using a mini-extruder (Avanti Polar Lipids) as previous literatures described. Finally, after stored at 4 °C over 2 h, the sample was subjected to ultracentrifugation at 100,000×g for 70 min at 4 °C, the resulting EVs was deposited in the bottom of ultra-centrifugal tube and then resuspended in normal saline for further research.

### Characterization of SVF-EVs

Morphology of SVF-EVs was characterized by a transmission electron microscopy (TEM, HT7800, HITACHI, Japan) as previous description^40^. The particle size distribution and concentration of SVF-EVs were assessed using Nanoparticle Tracking Analysis (NTA, NanoSight 300, Malvern Panalytical, UK) equipped with a blue laser (405 nm) as before described. The sample was diluted 10^3^ times with sterile normal saline and the quantities of diameter and concentration of SVF-EVs were displayed through image visualization.

### Western blotting

To detect the presence of tetraspanin proteins (TSG101 and CD81) in membrane, lipogenesis-related proteins (PPARγ and CEBPα) and angiogenic-related proteins (Ang-1 and VEGF) in the contents, proteins were harvested from SVF-EVs contents with RIPA Lysis Buffer (Beyotime, Hainan, Jiangsu, China) supplemented with phenylmethyl sulfonyl fluoride (PMSF) protease inhibitor. Denatured protein samples were subjected to sodium dodecyl sulfate polyacrylamide gel electrophoresis (SDS-PAGE) and then transferred to PVDF membranes. Then membranes were blocked with 5% skimmed milk, and incubated with specific antibodies against TSG101 (ab133586, Abcam, UK), CD81(ab109201, Abcam, UK), PPARγ (sc-7273, Santa, USA), CEBPα (sc-365318, Santa, USA), Ang-1 (23302-1-AP, Sanying, China) and VEGF (66828-1-Ig, Sanying, China) overnight at 4 °C. The membrane was then incubated with secondary antibodies which are purchased from GeneTex (Irvine, USA). Protein bands were visualized by the FluorChem Imaging System (ProteinSimple, San Jose, USA) using the Amersham Hyperfilm ECL reagent.

### Cellular uptake assay

PKH26 was used to label the fabricated particles following the manufacturer’s instructions. Human umbilical vein endothelial cells (HUVECs) were purchased from the National Collection of Authenticated Cell Cultures (China) and used as the recipient cells. HUVECs were incubated with PKH26-labeled SVF-EVs for 2 and 4 h in RPMI 1640 medium (Hyclone, USA) supplemented with 10% fetal bovine serum (FBS, Hyclone, USA) and 1% Penicillin–Streptomycin (PS, Servicebio, China), and washed three times with phosphate buffered saline (PBS, Servicebio, China). Then the cells were incubated with FITC-conjugated Anti-CD31 (Abcam, UK) overnight at 4 °C. After washed by PBS, cells were stained with 4′,6-diamidino-2-phenylindole (DAPI, Beyotime, China) and observed by a laser confocal microscope (A1Si, Nikon, Japan).

### Effects of SVF-EVs on HUVECs migration in vitro

The protein concentration of SVF-EVs was determined using BCA assay and then the fabricated vesicles were respectively added to the culture medium to a final protein concentration of 20 ng/mL and 40 ng/mL. HUVECs were seeded in 24-well plates at a density of 2 × 10^4^ cells/well and cultured until they reached a 90% confluence. Before scratching in the culture plate, HUVECs were starved and cultured in medium without FBS. Next, scratches were made on the cell surface layers using a pipette tip. The medium in each well were replaced with RPMI 1640 medium (without FBS) supplemented with 0 ng/mL, 20 ng/mL and 40 ng/mL SVF-EVs. The cell images in each well were captured using an optical microscope (Ts2, Nikon, Japan) at time 0, 6 and 12 h during culturing. The wound areas were quantified via measuring 5 wells of each group using Image-Pro Plus software (Media Cybernetics, USA) as previously reported^41^.

### Effect of SVF-EVs on adipogenesis of ADSCs in vitro

#### Isolation of ADSCs and oil red O staining

ADSCs were isolated from human adipose tissue and cultured in dulbecco's modified eagle medium (DMEM, Hyclone, USA) supplemented with 10% fetal bovine serum and 1% Penicillin–Streptomycin using the method described in previous report^40^. ADSCs were incubated with PKH26-labeled SVF-EVs for 2 h, then stained with calcein AM (Aladdin, China) and DAPI according to the kit’s instruction book, and observed by confocal microscope.

Then ADSCs were seeded into 12-well plate at a density of 2 × 10^4^ cell per well and grown to 50% confluence. The protein concentration of SVF-EVs was determined using BCA assay and then the fabricated vesicles were respectively added to the culture medium to a final protein concentration of 40 ng/mL. The control group was treated with the same volume of sterilized normal saline and the culture medium in three groups was changed every other day. Oil Red O staining was conducted at days 7 and 14 of culture according to the manufacturer’s instruction (Solarbio, China). The cells were washed again with distilled water and observed with an inverted aberration microscope (Ts2, Nikon, Japan). The quantitative statistics of Oil Red O positive area were quantified via measuring 5 images of each wells using Image-Pro Plus softwareas previously reported^42^.

#### qRT-PCR analysis

Total RNA was extracted from ADSCs cultured for 14 days in the 0 ng/mL and 40 ng/mL SVF-EVs group using SeraMir RNA purification kit (System Biosciences, USA). Primers used for qRT-PCR analysis was presented in Table 1 and the process was performed using the StepOnePlus Real-Time PCR System (Applied Biosystems, USA) as the manufacturer’s instruction.

**Table 1:** Primers used for qRT-PCR analysis.

Gene name	Forward sequence (5’-3’)	Reverse sequence (5’-3’)
β-actin	CACGATGGAGGGGCCGGACTCATC	TAAAGACCTCTATGCCAACACAGT
Homo PPARγ	GACCACTCCCACTCCTTTGA	ATGAGGGAGTTGGAAGGCTC
Homo FAS	CCGGACCCAGAATACCAAGT	GAAGACAAAGCCACCCCAAG
Homo adiponectin	GGAGTCGGAACATTGGCATC	TACCAAGGCATCCACGACTT

#### Western blotting

The total proteins of cells in the 0 ng/mL and 40 ng/mL SVF-EVs group were assessed by the BCA assay, and then separated by SDS-PAGE and transferred to PVDF membranes. Each blot was blocked with 5% skimmed milk, and incubated were immunodetected with specific antibodies against PPARγ (Santa, USA), FAS (Santa, USA), adiponectin (ADPN, Santa, USA), and GADPH (Antgene, China) overnight at 4 °C. The membrane was then incubated with secondary antibodies and the protein bands were visualized visualized by the FluorChem Imaging System (ProteinSimple, San Jose, USA) using the Amersham Hyperfilm ECL reagent.

### Preparation of DAT

DAT was prepared according to previous report^43^. Briefly, subcutaneous fat harvested via liposuction from donors at the Wuhan Union hospital and subjected to three freeze–thaw cycles. With washing and centrifuging at 1200×g for 5 min, the samples separated into three layers and the middle matrix layer was collected and then digested with 0.25% trypsin and 0.1% EDTA (Servicebio, China). After enzymatic digestion, the tissues were washed and subjected to polar solvent extraction using isopropanol at for 48 h 37 °C. The samples were subsequently treated with enzymatic digestion solution containing 20 ng/mL DNAse (Sigma, USA), and 20 ng/mL RNAse (Sigma, USA) for 16 h at 37 °C. Then, the processed tissues were rinsed with PBS 3 times and subjected to another 48 h’ extraction using isopropanol to remove reminded lipid. After several washes with PBS, DAT was collected and stored in absolute ethanol at 4 °C until needed.

Adipose tissue and prepared DAT were dehydrated via a graded alcohol series and embedded in paraffin. After cut the samples into sections in 5 μm thickness, the DAPI staining was performed to investigate the nucleus in tissues. Hematoxylin & Eosin (HE), Masson's Trichrome (Masson), and Oil Red O staining were conducted to distinguish the tissue structure changes between adipose tissue and DAT.

### Adhesion and viability of ADSCs in prepared DAT

The adhesion and viability of ADSCs in prepared DAT were evaluated according to the previous study^44^. Briefly, DAT particles were placed in 24-well plates and ADSCs were seeded on DAT at 2 × 10^4^ per 50 μL media and then kept in a cell incubator for 2 h. Subsequently, 1 mL DMEM media was added into the well and changed every other day. To evaluate the adhesion and viability of ADSCs in prepared DAT, the cell-DAT constructs were stained using a Live/Dead assay at 1, 4, and 7 days of culture. Observing by the confocal microscopy, living cells were stained with fluorescein diacetate (FDA, Sigma) and dead cells were stained with propidium iodide (PI, Sigma). The integrated optical density (IOD) was calculated by Image-Pro Plus software.

### Animal study

All mice used in this study were purchased from the animal center of Huazhong University of Science and Technology (HUST) and the experimental protocols concerning animal were approved by the Animal Ethical Committee of HUST. In this study, animal experiments were conducted under the supervision of the Animal Center of HUST and in accordance with relevant guidelines and regulations. Four-week-old male BALB/c nude mice were fed in the specific pathogen-free animal facilities and maintained under conventional conditions.

10 mL prepared DAT was trimmed into microparticles which could be injected with 18-G needles and SVF-EVs were respectively added to the injectant to a final protein concentration of 40 ng/mL. Twelve nude mice were randomly divided into two groups (0 ng/mL and 40 ng/mL SVF-EVs) and then injected 0.3 mL of injectant into their dorsal region. Mice were sacrificed by cervical vertebra dislocation at 4 weeks and 8 weeks’ post-injection, and the grafts were harvested and weighed for volume.

The harvested samples were dehydrated via a graded alcohol series and embedded in paraffin. After cut the samples into sections in 5 μm thickness, HE staining was conducted to distinguish the tissue structure. Sections used for immunofluorescent staining were incubated with primary perilipin (Abcam, UK), CD31 (Abcam, UK) antibody and subsequently respectively incubated with FITC- and PE- conjugated goat anti-rabbit IgG antibody (Servicebio, China). Nuclei were counterstained with DAPI stain. Moreover, a CD31immunohistochemical staining was performed according to the manufacturer’s protocol (Yaji Bio, China). The images of histological analysis were captured by a Nikon Ni microscope.

### Statistical analysis

All data collected in this work were expressed as mean ± SD and analyzed with SPSS 21.0 software (IBM, USA). A Student’s t test was used for the comparisons between two groups and one-way analysis of variance (ANOVA) test with post hoc contrasts by Newman–Keuls test was used to analyze differences in multiple comparisons. *P* < 0.05 was considered to be a statistically significant difference.

### ARRIVE guidelines

This study is conducted in accordance with ARRIVE guidelines.

## Results

### Characteristics of SVF-EVs

**Figure 1:**
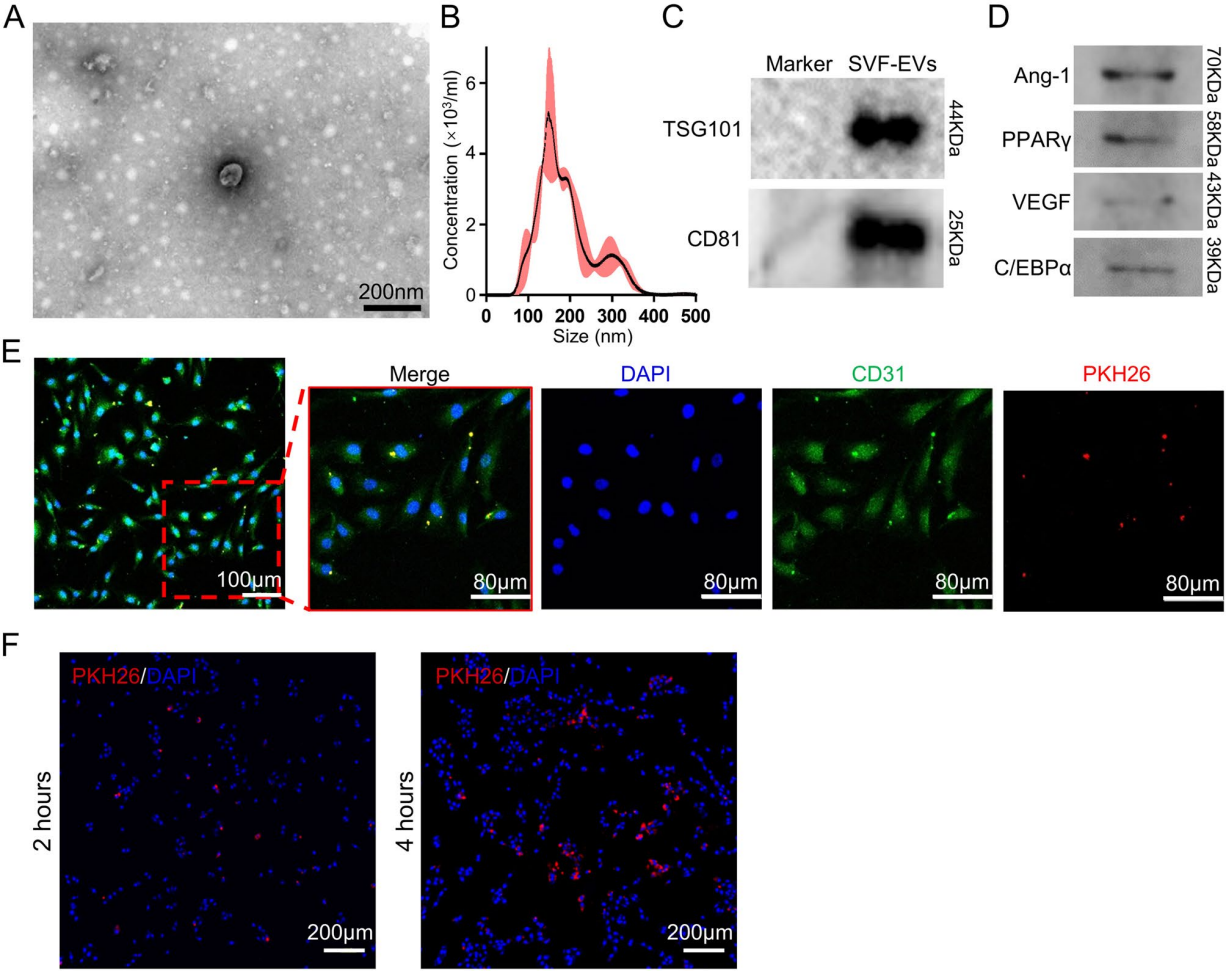
Characteristics of SVF-EVs. (**A**) TEM images of SVF-EVs. (**B**) NTA analysis of SVF-EVs. The confirmed size distribution with a range of diameter around 100–300 nm. (**C**) Western blot shows that SVF-EVs express tetraspanin proteins like TSG101 and CD81. (**D**) Western blot reveals that SVF-EVs encapsulate proteins such as PPARγ, C/EBPα, Ang-1, and VEGF. (**E**) Confocal images verify the internalization of PKH26-labeled SVF-EVs (red) into endothelial cell (CD31-labeled, green), and the amount of endocytosed SVF-EVs after co-culturing for 4 h is more than that for 2 h (**F**).

Analysis of TEM images (Fig. 1A) illustrated that SVF-EVs displayed 100 nm spherical vesicles enclosed by a bilayer lipid membrane. The NTA of SVF-EVs (Fig. 1B) confirmed the size distribution with a range of diameter around 100–300 nm. Western blot analysis revealed that SVF-EVs express tetraspanin proteins (Fig. 1C), such as CD81 and TSG101, and some proteins (Fig. 1D), such as PPARγ, C/EBPα, Ang-1 and VEGF. After 2 h of co-culture (Fig. 1E), the PKH26-labeled SVF-EVs (red) were internalized into CD31-labeled HUVECs (green) and surrounded the nucleus (blue). And with the increase of co-culture time, the concentration of engineered vesicles inside cells increased and showed stronger red fluorescence under confocal microscope (Fig. 1F).

### Effects of SVF-EVs on HUVECs migration in vitro

**Figure 2:**
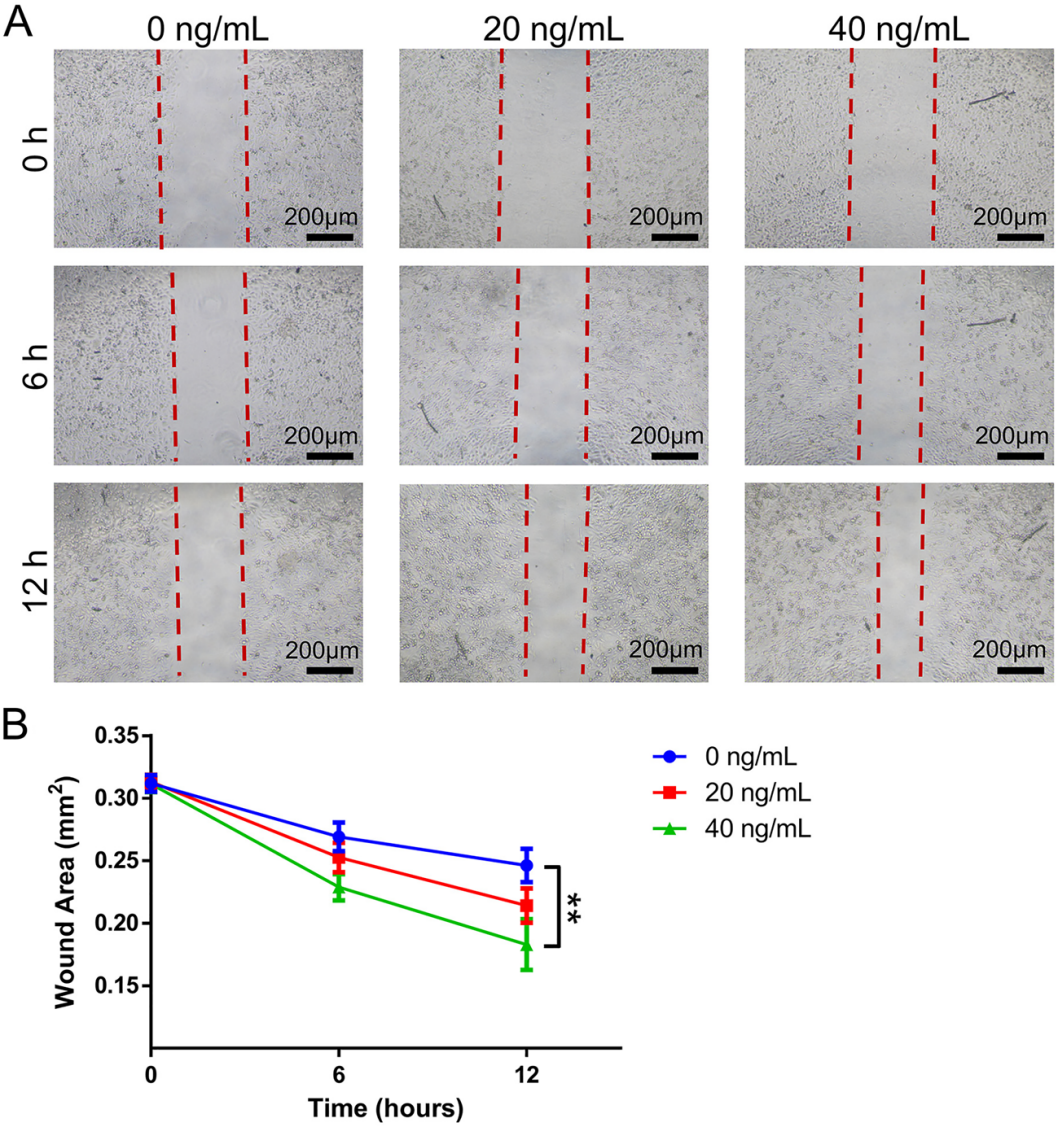
Effects of SVF-EVs on HUVECs Migration in vitro*.* (**A**) Images from scratch experiment for three groups (0, 20, 40 ng/mL proteins encapsulated in SVF-EVs) at different time points. (**B**) The quantitative statistics of residual wound area in three groups at different time points (**: *p* < 0.01, n = 5).

The general HUVECs migration trend for the three groups is shown in Fig. 2A, SVF-EVs at 40 ng/mL exhibit a more obvious effect of promoting HUVECs migration than that with no SVF-EVs or low concentration of SVF-EVs (20 ng/mL). The wound area in three groups are calculated and the result is shown in Fig. 2B. The quantitative analysis reveals that the residual wound area of the 40 ng/mL SVF-EVs group at the 12th hour (182.89 ± 20.39 × 10^−3^ mm^2^) was less than that of the 20 ng/mL SVF-EVs group (214.15 ± 13.76 × 10^−3^ mm^2^) and blank group (240.16 ± 13.85 × 10^−3^ mm^2^), and the difference has statistically significant (*p* < 0.01, n = 5).

### Effects of SVF-EVs on adipogenic differentiation of ADSCs in vitro

**Figure 3:**
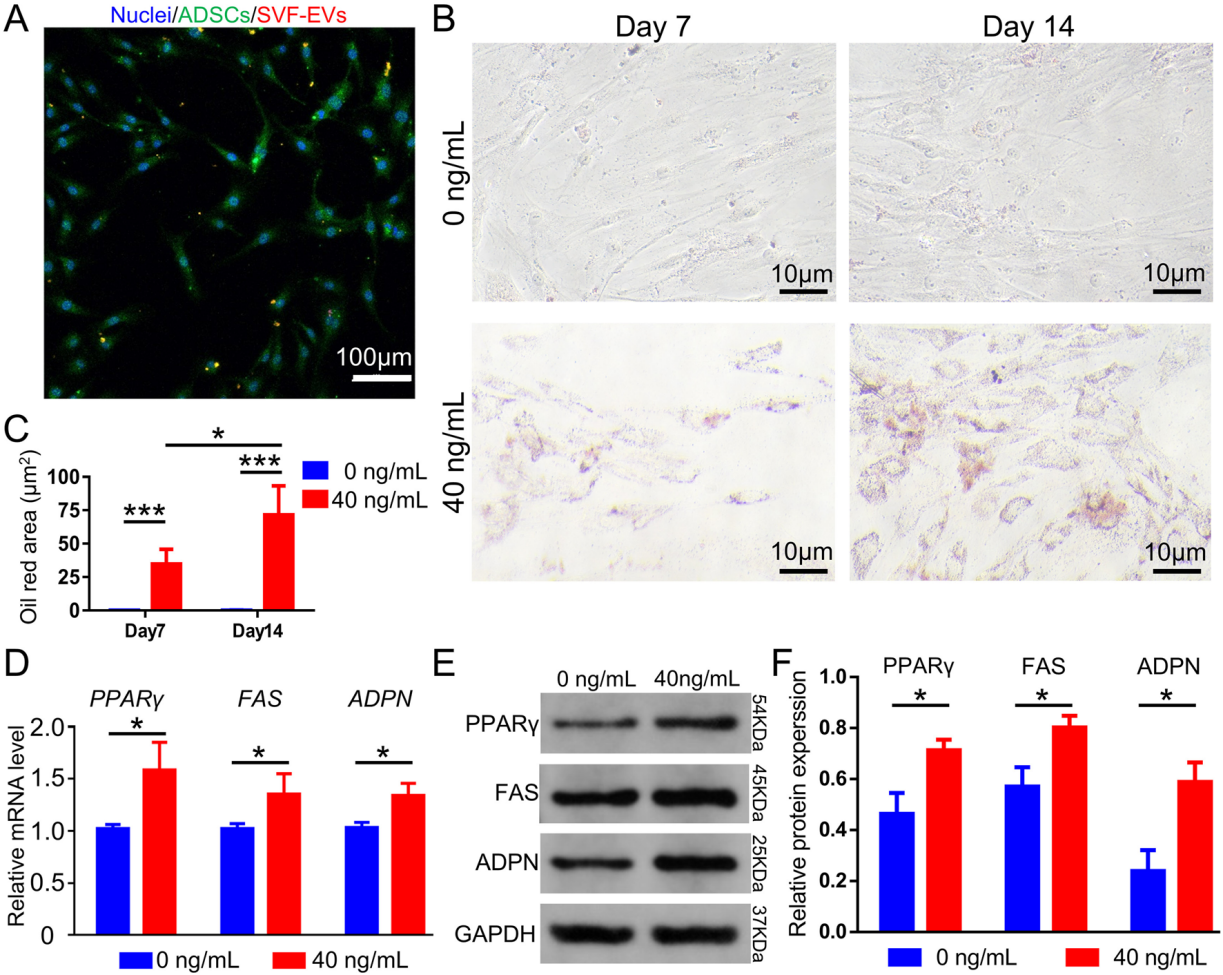
Effects of SVF-EVs on lipogenic Differentiation of ADSCs in vitro. (**A**) Confocal microscopy image shows that ADSCs (green) endocytose PKH26-labeled SVF-EVs (red). (**B**) Images from Oil Red O staining for two groups (0 and 40 ng/mL proteins encapsulated in SVF-EVs) at different time points. (**C**) The quantitative statistics of Oil Red O positive area in two groups at different time points (*: *p* < 0.05, ***: *p* < 0.005, n = 5). (**D**) The qRT-PCR analysis of mRNA expression of lipogenic genes such as PPARγ, FAS, and ADPN in ADSCs (*: *p* < 0.05, n = 3). The western blot (**E**) and quantitative analysis (**F**) of the adipogenic-associative protein levels in ADSCs, reveals that the SVF-EVs promote lipogenic differentiation of ADSCs (*: *p* < 0.05, n = 3).

Confocal microscopy images (Fig. 3A and S1) illustrate that PKH26-labeled SVF-EVs (red) were endocytosed by ADSCs (green). Images (Fig. 3B) from the Oil Red O staining and quantitative statistics of Oil Red O positive area (Fig. 3C) show that SVF-EVs promote lipids synthesis in ADSCs, and the amounts of synthetic lipid are correlated with the co-cultured time. To further investigate the mechanisms of adipogenesis in vitro, qRT-PCR analysis (Fig. 3D) was performed and showed that the mRNA expression of lipogenic genes such as PPARγ, FAS, and ADPN in ADSCs co-cultured with 40 ng/mL SVF-EVs are higher than that without SVF-EVs. Western blot (Fig. 3E) and quantitative analysis of optical density (Fig. 3F) verified that the protein levels of PPARγ, FAS, and ADPN are significantly increased in ADSCs treated with SVF-EVs.

### Characteristics and histological investigation of the prepared DAT

**Figure 4:**
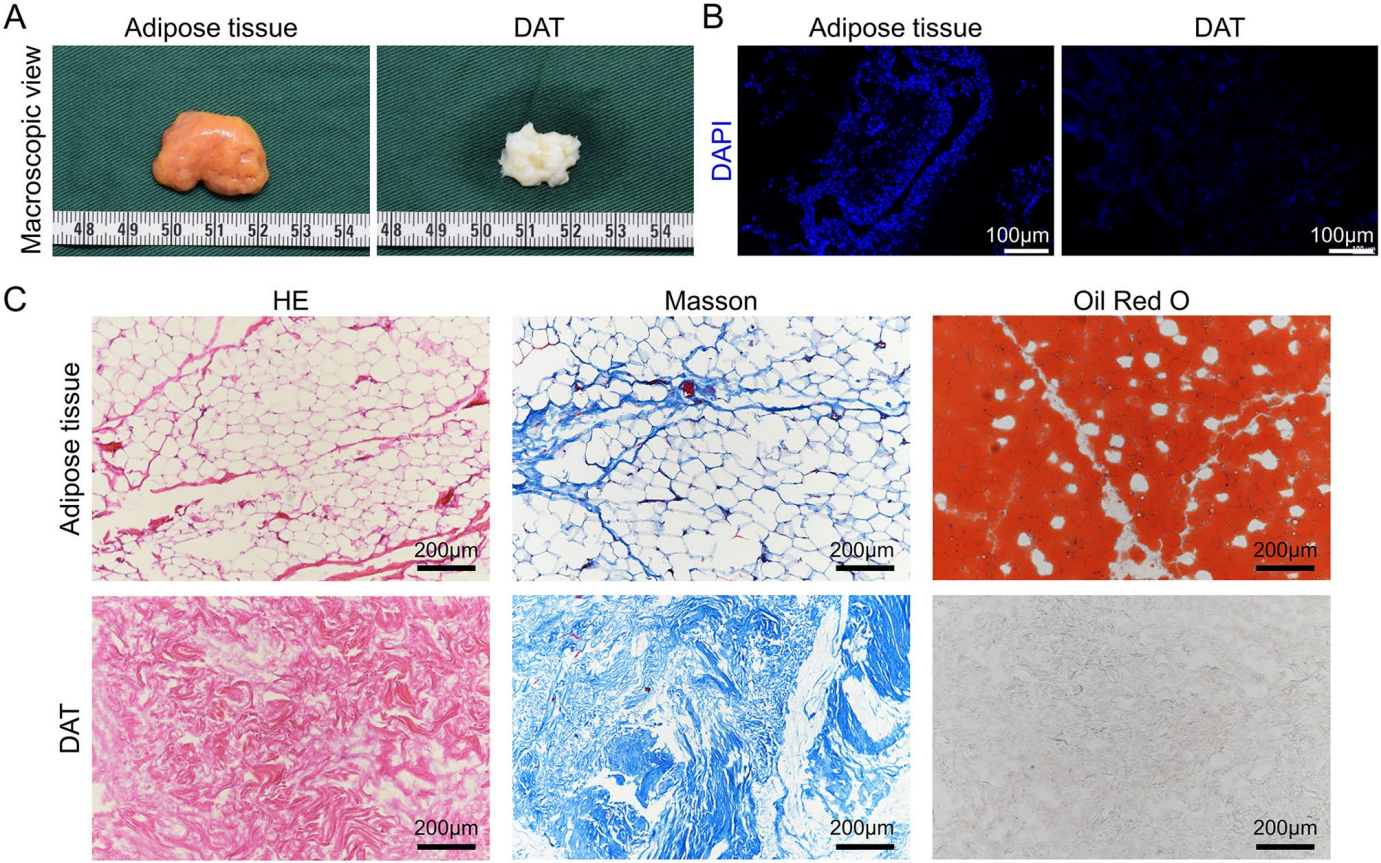
Characteristics of DAT. (**A**) The macroscopic view of adipose tissue and prepared DAT. (**B**) Images of DAPI fluorescence staining of adipose tissue and prepared DAT. (**C**) Images of HE, Masson, and Oil Red O analysis of adipose tissue and prepared DAT.

DAT appeared an ivory white in contrast to the yellow appearance of adipose tissue (Fig. 4A). After the decellularization progress, DAPI staining (Fig. 4B) demonstrated that DAT was cell-free and histological analysis (Fig. 4C) revealed the DAT was mainly composed of collagen and was disordered due to the loss of the compact arrangement structure of adipocytes. Oil Red O staining showed that the lipid was also completely removed after decellularization.

### Biocompatibility evaluation of the prepared DAT

**Figure 5:**
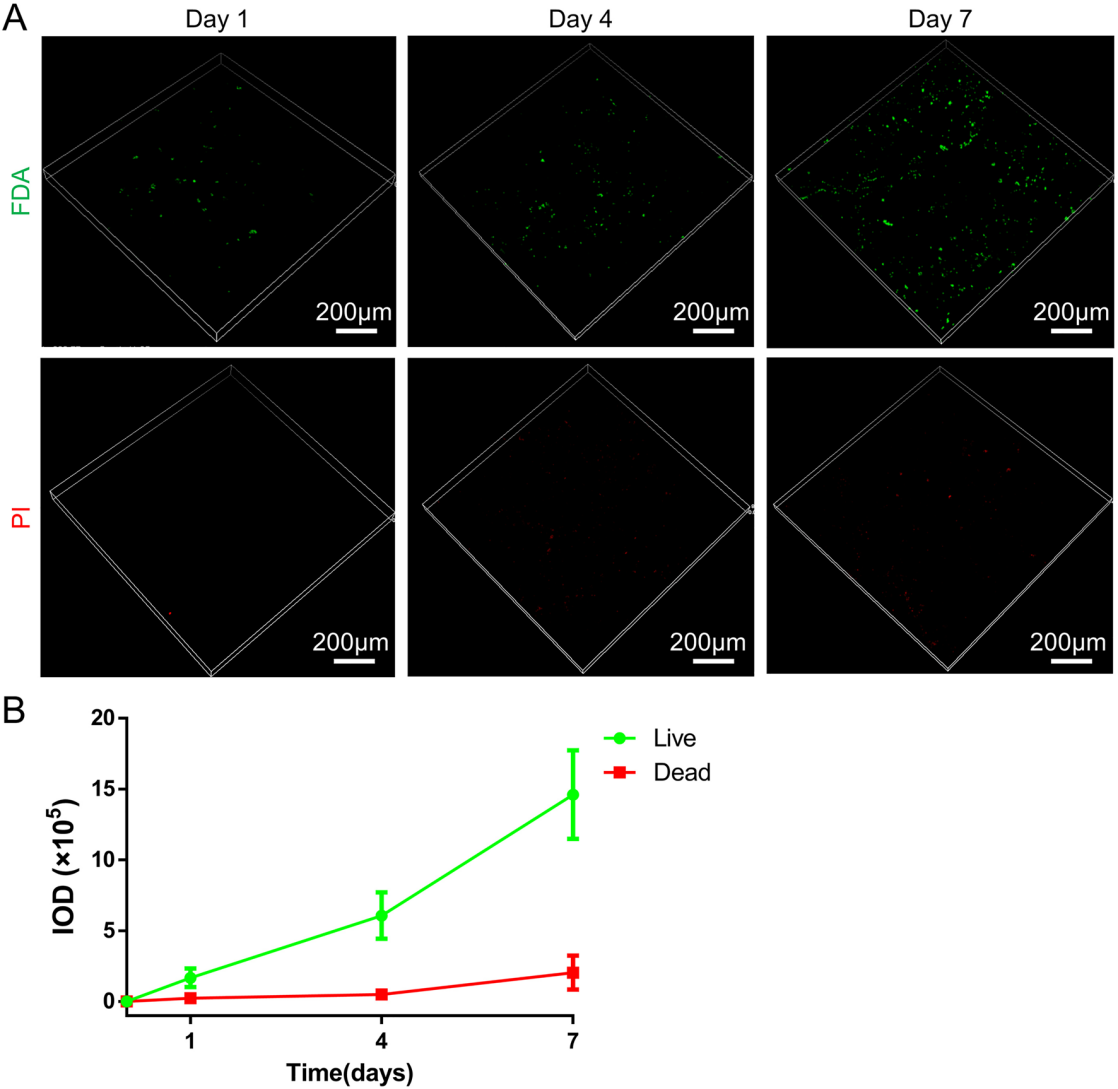
Biocompatibility evaluation of DAT. (**A**) Confocal fluorescent images for FDA/PI staining of ADSCs cultured on the DAT for 1, 4, and 7 days. (**B**) The quantitative results of the relative integral optical density (IOD) of FDA and PI labeled ADSCs, and this trend exhibits the acceptable biocompatibility of DAT.

The live/dead staining of ADSCs at day 1, 4 and 7 (Fig. 5A) showed that a large number of living cells (green) could be seen in the prepared DAT, indicating that ADSCs have good viability on the acellular matrix. Meanwhile, the number of dead ADSCs (red) remained low and did not change significantly with the increase of culture time. Figure 5B was the quantitative results of the relative integral optical density of FDA and PI labeled cells.

### In vivo study

**Figure 6:**
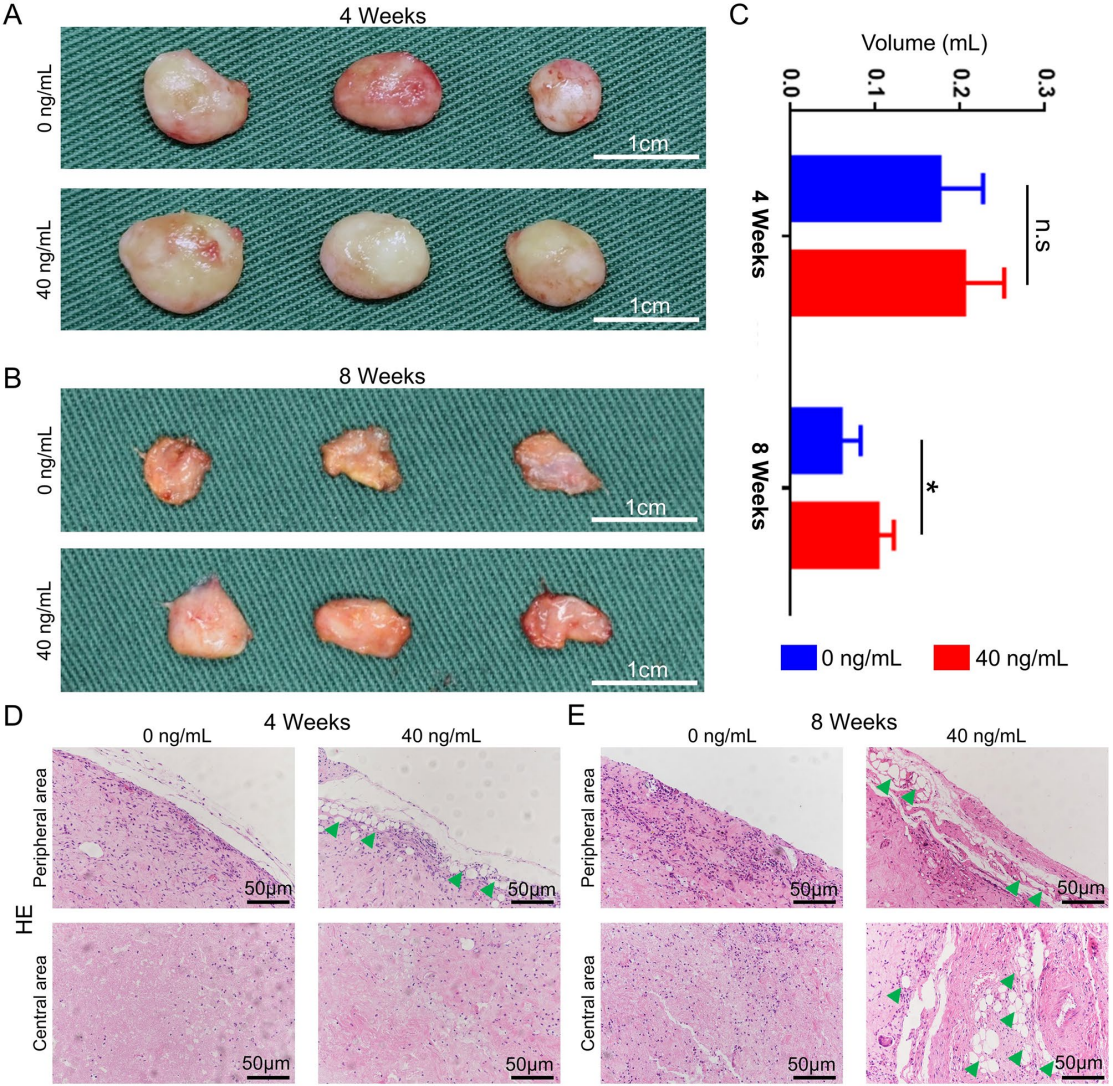
Harvested sample evaluation of transplanted DAT along with SVF-EVs. Macroscopic images of the DAT grafts mixed with 0 ng/mL or 40 ng/mL SVF-EVs after 4 weeks (**A**) and 8 weeks (**B**) transplantation. (**C**) The quantitative analysis (n = 3) of the volume of subcutaneous samples after transplanted for 4 and 8 weeks (*: *p* < 0.05, n.s: no significance.). The representative images of HE staining at the peripheral and central area of harvested samples after transplanted for 4 weeks (**D**) and 8 weeks (**E**) (green triangle labels vacuolated structure).

Figure 6A and B respectively showed the macroscopic images of the grafts after subcutaneous transplanted in nude mouse for 4 weeks and 8 weeks. The grafts presented a soft tissue appearance with a complete capsule, and the volume of grafts after transplanting for 8 weeks decreased significantly compared with that at 4 weeks. After 4 weeks of subcutaneous transplantation of DAT along with SVF-EVs (40 ng/mL), the volume of harvested grafts was 205.41 ± 47.34 mm^3^ compared to 176.33 ± 51.50 mm^3^ in the blank (0 ng/mL) group, which was no statistically different between the two groups (Fig. 6C). But after transplanted for 8 weeks, the volume of grafts in SVF-EVs was 103.81 ± 19.18 mm^3^ and that in blank group was 60.33 ± 23.71 mm^3^, the difference between the two groups was statistically significant (*p* < 0.05, n = 3).

After 4 weeks of transplantation (Fig. 6D), there was obvious cell infiltration in the peripheral area of DAT, while the density of cells entering the central area of DAT was less. In the 40 ng/mL SVF-EVs group, representative graft images showed some continuous vacuolated structures (green triangle) appeared at the graft edge. Furthermore, when HE staining was performed on grafts eight weeks after transplantation (Fig. 6E), we found that such vacuolated structures increased in numbers in the 40 ng/mL SVF-EVs group, and vacuolated structures were present not only at the periphery of the grafts, but also at the central area.

**Figure 7:**
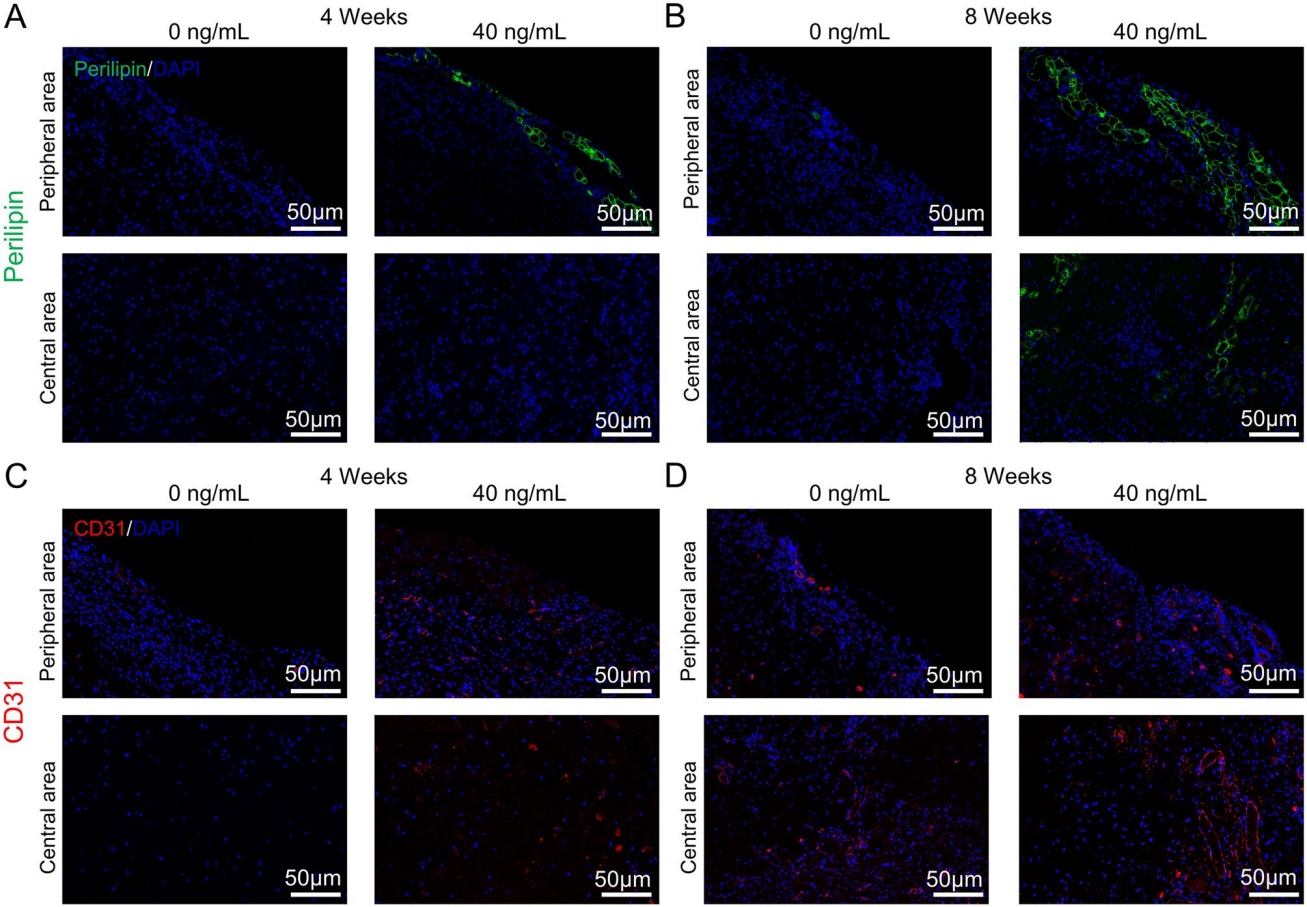
Immunofluorescent staining for angiogenesis and adipogenesis evaluation in DAT. The representative images of immunofluorescent staining for perilipin at the peripheral and central area of grafts after transplanted for 4 weeks (**A**) and 8 weeks (**B**). (**C**, **D**) The representative images of immunofluorescent staining for CD31 at the peripheral and central area of grafts after transplanted for 4 weeks and 8 weeks.

To further clarify the structures of the harvested grafts, we further performed immunofluorescent staining for perilipin and CD31. The results (Fig. 7A,B) found that the vacuolated structures can be labeled with green perilipin fluorescence and indicate adipocytes. Compared with the blank group, grafted with 40 ng/mL SVF-EVs promoted the formation of adipocytes in DAT, and this effect was significant over time. CD31 immunofluorescent staining images (Fig. 7C,D) exhibited that a large number of new blood vessels with significantly larger lumen diameter were formed in the central area of DAT after 8 weeks’ transplantation with 40 ng/mL SVF-EVs. CD31 immunohistochemical staining images (Fig. S2) showed the morphology of new vessels, with red blood cells visible inside.

## Discussion

Vascularization is a critical physiological step for survival and regeneration after tissue transplantation, and inadequate vascularization in practice has led to many clinical problems that are difficult to resolve. Lack of vascularization in the center of chronic wounds leads to slow growth of granulation tissue and difficult healing, insufficient perfusion of the distal part of the flap after transplantation causes vascular crisis, and the lack of blood supply to the follicular unit transplanted in the scar area appears an infertile survival. In this study, we prepared SVF as nanoscale vesicles and proved its promotion of vascularization in DAT and inducing adipocyte regeneration. The experimental results show that SVF-EVs has the potential to be an option to address the vascularization deficit.

SVF has been shown in many studies to have a pro-vascularizing and thus fat regenerating effect^45,46^. Zhong et al.^45^ mixed SVF with free adipose tissue and then autografted it to increase adipocyte survival and regeneration rates. Lu and his colleagues prepared SVF into SVF-gel to achieve better restorative results in facial contour remodeling and rejuvenation^46^. In this paper, we prepared SVF in the form of nanovesicles and investigated its effect on promoting the revascularization of the acellular matrix, enriching the ways SVF can be applied, thereby increasing the time interval for SVF to maintain its biological effect after derived from adipose tissue. Through in vitro experiments, we found that SVF prepared in vesicle form promotes adipocyte formation and enhance vascular endothelial cell migration biological effects, and we hypothesize that these are closely related to the protein encapsulated in the vesicles. The transcription factor, PPARγ, plays a key role in adipocyte formation, and pro-adipocytes initiate lipogenic differentiation in response to PPARγ, with increased expression of intracellular fatty acid synthase (FAS), becoming mature adipocytes and secreting representative hormones such as adiponectin^47,48^. Ang-1 promotes endothelial cell outgrowth and migration, and VEGF promotes endothelial cell proliferation and the formation of lumen structures, which play an important role in the revascularization of the acellular matrix^49^. In our study, western blot detected these key lipogenic and angiogenic-associated growth factors in vesicles. Meanwhile, some miRNAs, lncRNAs, and other nucleic acids have been shown to regulate adipogenesis and angiogenesis, and engineered vesicles may also be encapsulated with these molecules, and this aspect is worth further investigation^50^.

After migration of endothelial cells into the acellular matrix, the cell morphology gradually forms vacuole, and multiple endothelial cells together form a lumen, with branching and remodeling, eventually forming a vascular network^51^. During this angiogenic process, the vascular lumen diameter gradually increases, and the structure of the formed lumen becomes more stable^52^. We performed CD31 immunofluorescence staining of grafts harvested from the animal study and showed that the central region of the graft in the SVF vesicle group showed relatively large tubular neovascularization at eight weeks, whereas only a small cellular vacuole formed inside the control group, and demonstrated a more stable, mature neovascular network formed inside the DAT in the SVF vesicle group. This is consistent with the results of in vitro experiments that SVF vesicles have a pro-angiogenic biological effect and promote revascularization of the acellular matrix.

ADSCs are adipose tissue-derived mesenchymal stem cells that have the potential to differentiate into osteogenesis, adipogenesis, and chondrogenesis but require the addition of induction factors to differentiate in a specific direction when cultured in vitro^28^. In this study, ADSCs were cultured with complete medium supplemented with 40 ng/mL SVF-EVs or not for 7 and 14 days. Particularly, ADSCs co-cultured without SVF-EVs had no intracellular lipid droplet formation after 14 days of culture, which is consistent with the fact that adipogenic differentiation of ADSCs requires the addition of adipogenic inducers like dexamethasone, insulin, and indomethacin in the culture medium. This results reminded us that SVF-EVs can induce adipogenic differentiation of ADSCs in vitro.

The metabolic processes of lipid storage and lipolysis of adipocyte require high oxygen consumption^53^. Hence, the rich vascular network within the adipose tissue and the degree of re-vascularization after autologous fat grafting has an important impact on volume retention^42,54^. The DAT contains more cytokines that promote adipose tissue formation and is more conducive to adipose tissue regeneration than other tissues^54^. Histological staining such as HE, Masson, and Oil Red O staining showed that the DAT lost the inherent adipocytes and vascular structures of adipose tissue, retaining only the loosely arranged extracellular matrix and luminal structures. In this study, DAT was transplanted into the subcutaneous of nude mice, and volume retention of the acellular matrix was maintained at more than 60% after four weeks. After eight weeks of transplantation, volume retention declined to approximately 20%, and the addition of SVF-EVs to promote revascularization of the acellular matrix was maintained at 30%. We suggest that the low volume retention of grafts after transplantation is related to the relatively sparse nature of the DAT but the low tissue elastic modulus facilitates regeneration of adipose tissue in the acellular matrix.

The SVF-EVs were observed to possess the biological activity of promoting neovascularization and adipocyte regeneration in vitro and in vivo, but there still exist limitations in this research and characteristics of SVF-EVs worth further exploration. Limited number of samples may make the results less convincing and the accuracy affected. Moreover, no significant differences between 0 and 40 ng/mL SVF-EVs group was observed on histological images at 4 weeks, which not enough to show the SVF-EVs’ efficacy in promoting angiogenesis and adipogenesis. In addition, when we explored the contents of SVF-EVs, no quantitative method (like ELISA) was performed to quantify the proteins in the whole vesicle, which limited the interpretation of the mechanism related to SVF-EVs’ effect on promoting adipocyte regeneration and neovascularization in this study. We hold that this paper explores the feasibility of direct preparation of vesicles from biological tissues, namely SVF, and preliminary study of the effect of SVF-EVs on cell behavior. And further studies are needed to clarify the mechanism of growth factors action and promote the wide application of engineered nanovesicles.

## Conclusion

In summary, the engineered vesicle made from SVF demonstrated accelerating endothelial cell migration and promoting ADSC lipogenic differentiation in vitro. After transplanting for 8 weeks, more angiogenesis in the central region of the implant was observed using DAT grafting in conjunction with SVF-EVs. Our findings suggest that SVF-EVs can promote vascularization of acellular matrix and that the associated physical preparation for processing biological samples is feasible.

## Supplementary Information

Supplementary Information.

## Data Availability

All data included in this study are available upon request by contact with the corresponding author.
