# Supplementary material for: Engineered nanovesicles from stromal vascular fraction promote angiogenesis and adipogenesis inside decellularized adipose tissue through encapsulating growth factors
Source: Sci Rep. 2023 Jan 13;13:750. doi: 10.1038/s41598-022-27176-w (PMC9839776; doi:10.1038/s41598-022-27176-w)
Supplement: Supplementary file 1 — Supplementary Information. [file 41598_2022_27176_MOESM1_ESM.pdf]

Supplementary material

Engineered Nanovesicles from Stromal Vascular Fraction Promote Angiogenesis and Adipogenesis inside Decellularized Adipose Tissue through Encapsulating Growth Factors

Jun Tu, Yuyang Zeng, Ran An, Jiaming Sun, Huicai Wen

Original images of western blot

1. Original images of western blot data in Figure 2C: the macroscopic view of membranes that cut and then hybridization with specific antibodies.

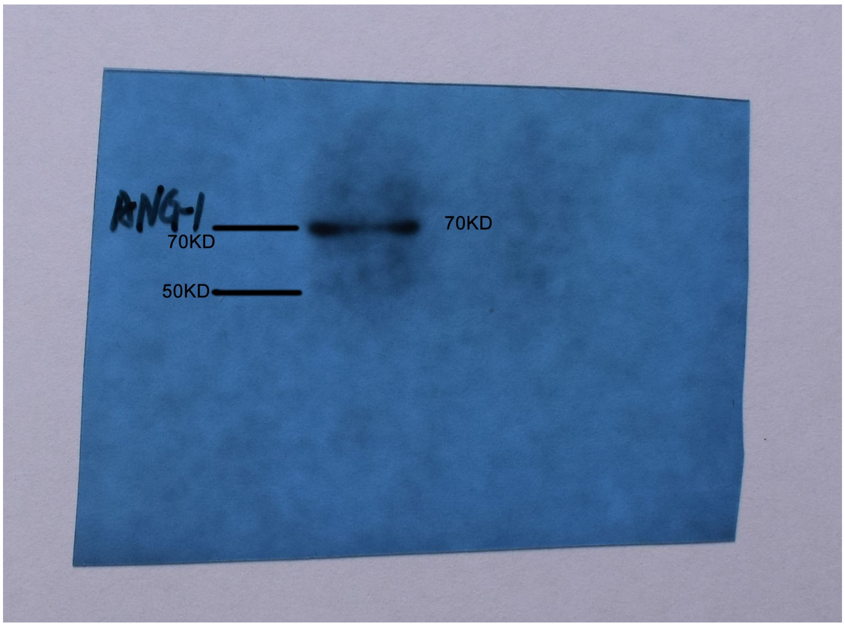

Ang-1

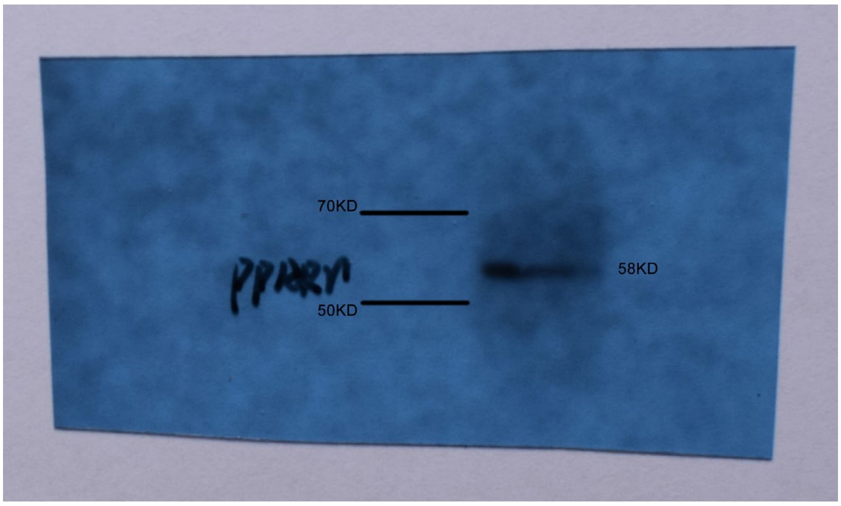

PPARγ

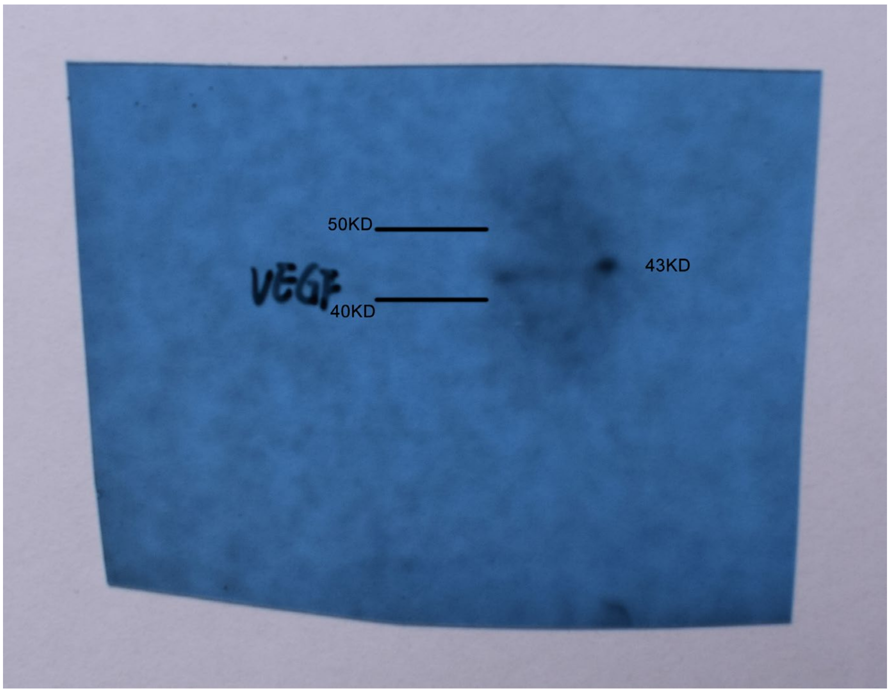

VEGF

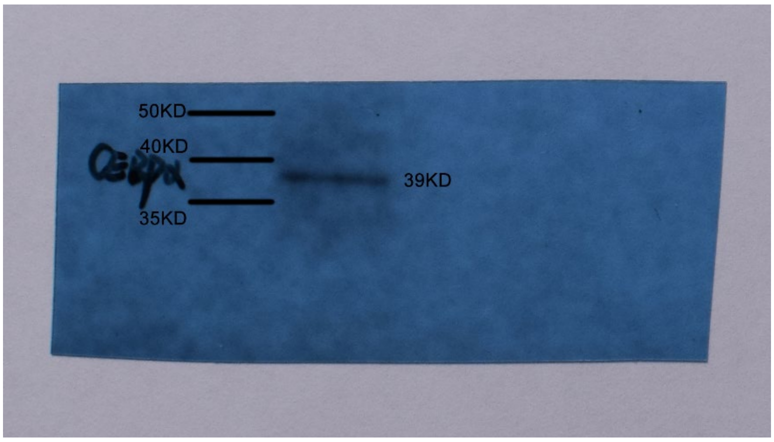

C/EBPα

2. Original images of western blot data in Figure 4D (repeat three times)

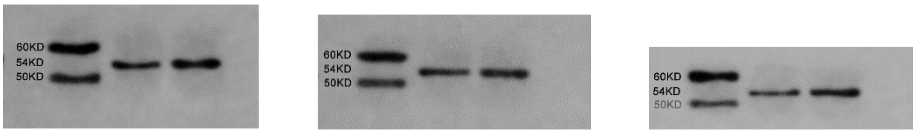

PPARγ

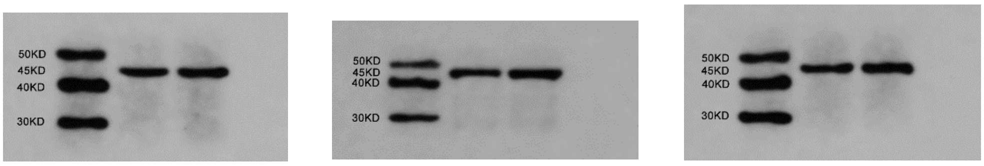

FAS

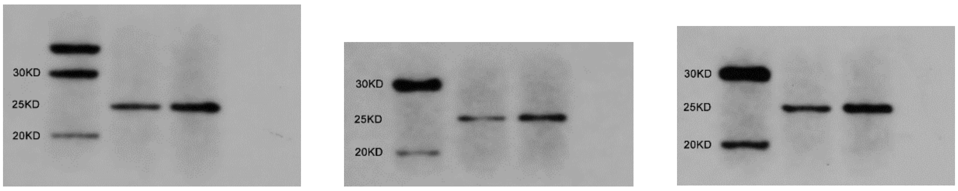

ADPN

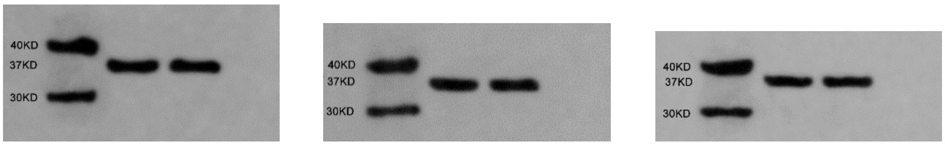

GAPDH

3. Original images of western blot data about tetraspanin proteins.

TSG101

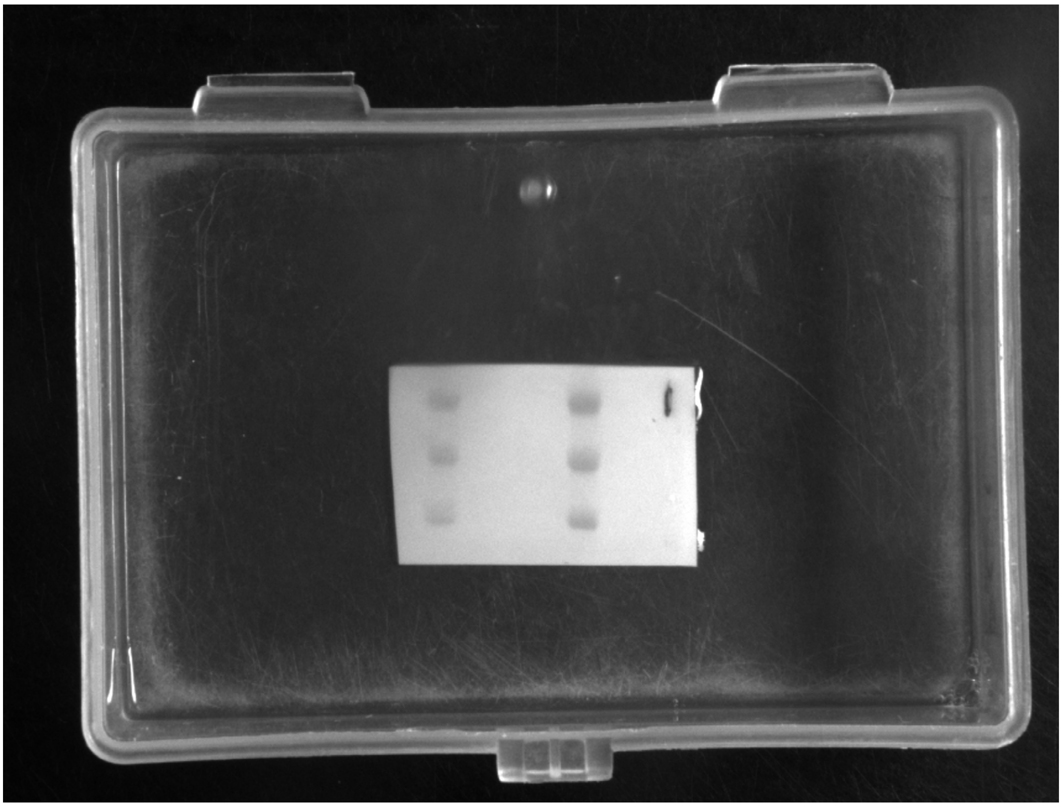

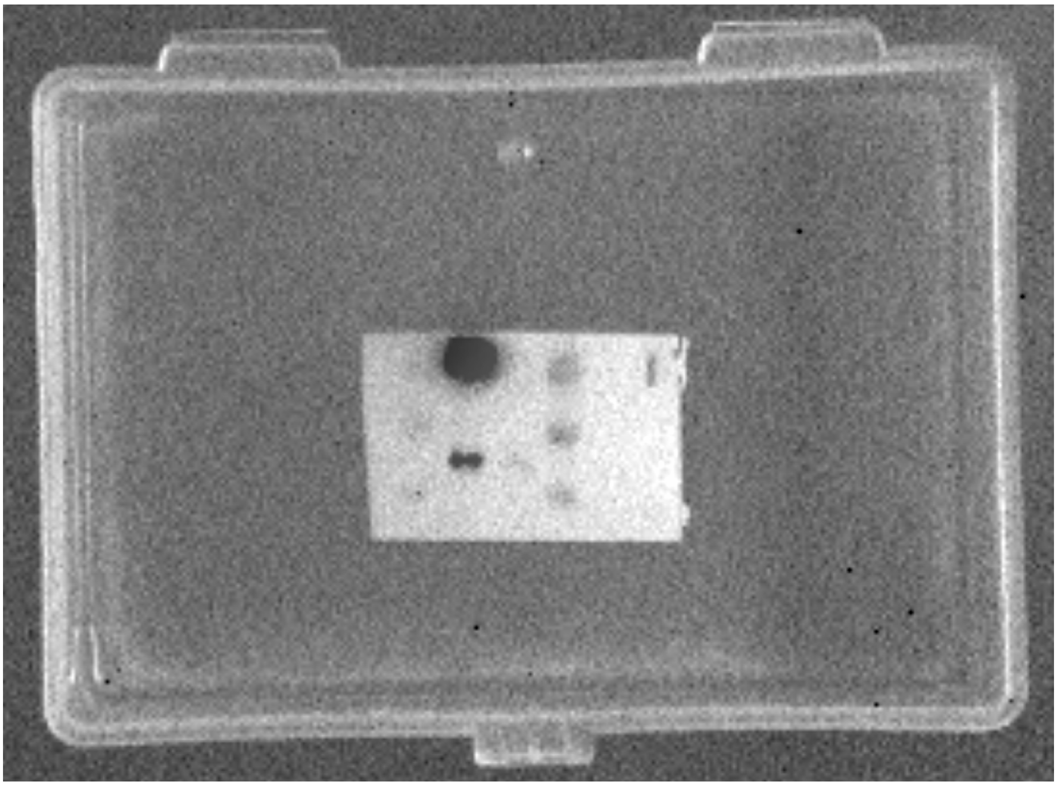

CD81

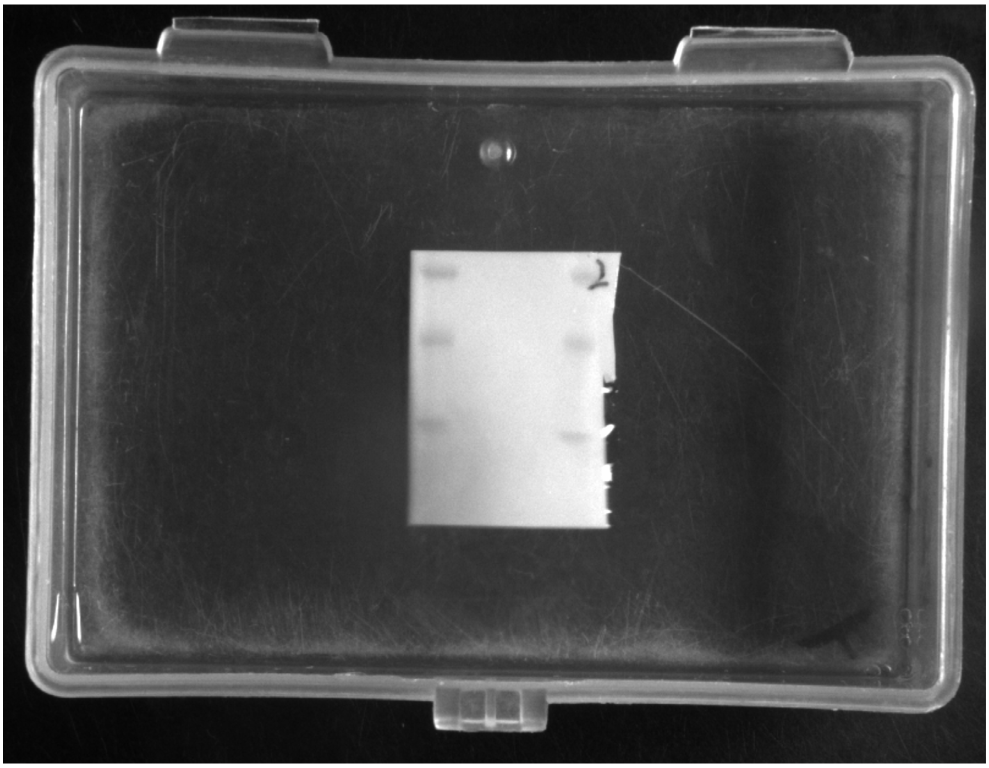

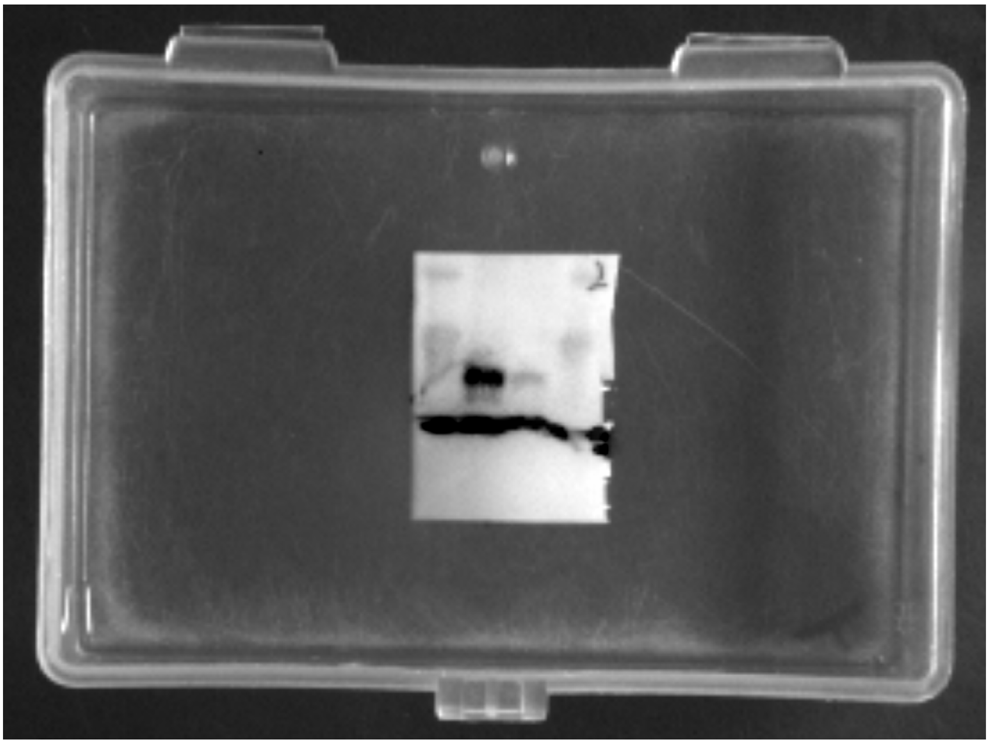

Supplementary figures

Figure S1

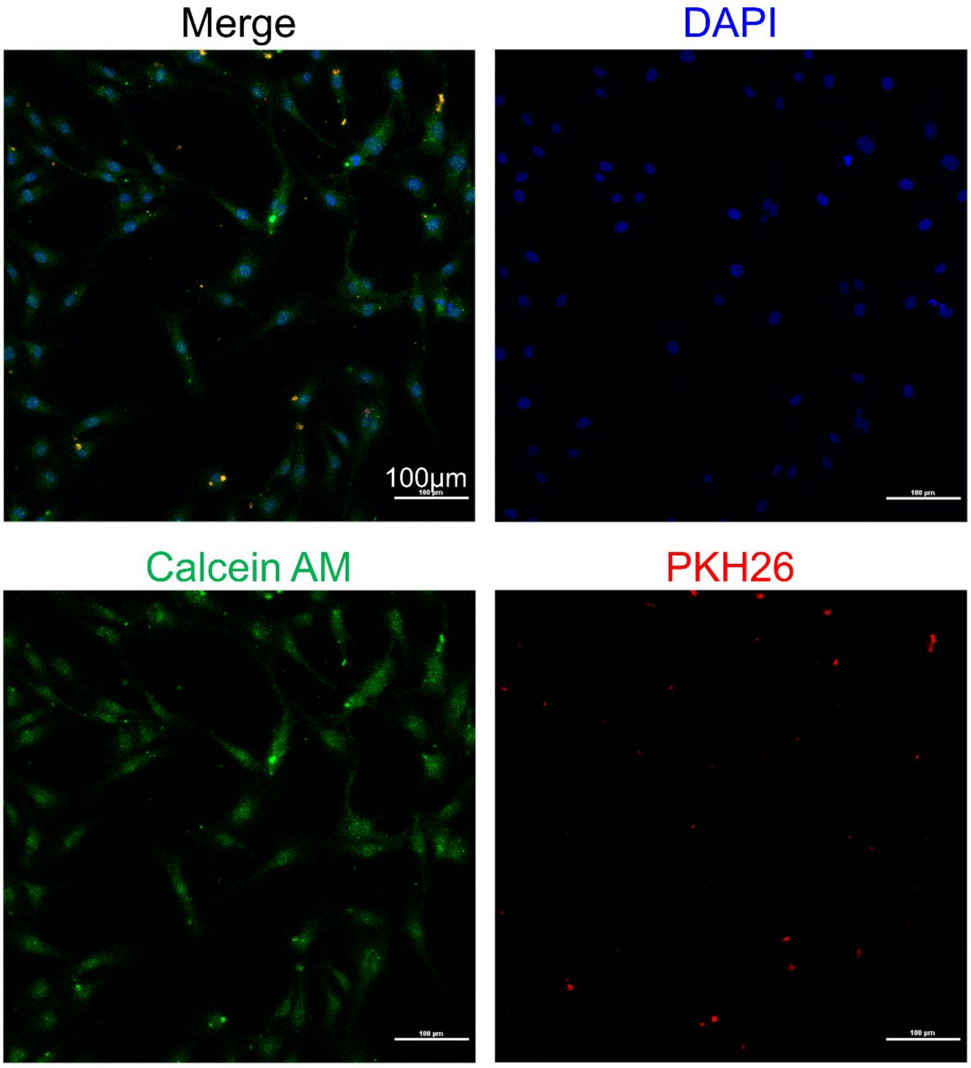

Figure S1. Confocal microscopy images of SVF-EVs (PKH26-labeled) endocytosed by ADSCs (Calcein AM stained).

Figure S2

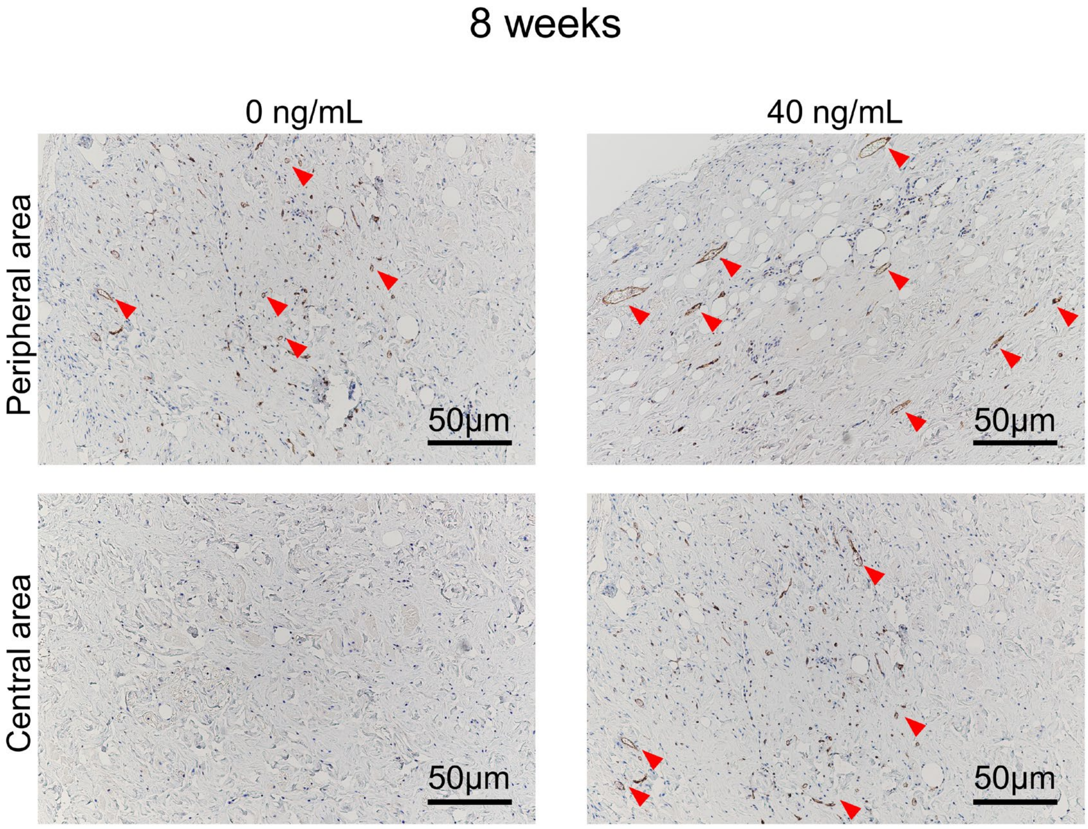

Figure S2. The representative images of CD31 immunohistochemical staining at the peripheral and central area of harvested samples after transplanted for 8 weeks. (arrow: new vessels)
